# Pharmacological Effects of the Water Fraction of Key Components in the Traditional Chinese Prescription Mai Tong Fang on 3T3-L1 Adipocytes and *ob/ob* Diabetic Mice

**DOI:** 10.3390/molecules190914687

**Published:** 2014-09-16

**Authors:** Liang Ma, Li Huang, Heying Pei, Zhuowei Liu, Caifeng Xie, Lei Lei, Xiaoxin Chen, Haoyu Ye, Aihua Peng, Lijuan Chen

**Affiliations:** 1 State Key Laboratory of Biotherapy and Cancer Center/Collaborative Innovation Center of Biotherapy, West China Hospital, West China Medical School, Sichuan University, Chengdu 610041, Sichuan, China; E-Mails: Liang_m@scu.edu.cn (L.M.); huangli_0709@126.com (L.H.); phy_03@sina.com (H.P.); xiecaifeng2013@163.com (C.X.); scuchemll@sina.com (L.L.); peng_aihua@163.com (A.P.); 2 Guangdong ZhongSheng Pharmaceutical Co. Ltd., Dongguan 440100, Guangdong, China; E-Mails: liuzhuowei@zspcl.com (Z.L.); chenzhenyu2000@zspcl.com (X.C.); 3 Institute of Translational Medicine, Nanchang University, Nanchang 330031, Jiangxi, China

**Keywords:** Mai Tong Fang (MTF), 3T3-L1 adipocyte, *ob/ob* mice, anti-adipogenic effect, anti-hyperglycemic activity

## Abstract

Mai Tong Fang (MTF), a Chinese herbal combination, has been used for the treatment of diabetic nephropathy in traditional medical clinics in China. However, the anti-adipogenic and anti-hyperglycemic effects of MTF have not been fully elucidated, so this study explored these pharmacological activities in 3T3-L1 adipocytes and *ob/ob* mice, respectively, of the water fraction of milkvetch root, salviae miltiorrhizae and mulberry as key components of MTF. MTF was found to inhibit adipogenesis and triglyceride accumulation in 3T3-L1 adipocytes. Oral administration of MTF in *ob/ob* mice for 8 weeks, exhibited positive controls on blood glucose and body weight, and further improved glucose tolerance according to an oral glucose tolerance test. Importantly, MTF extract alleviated fat deposition and ballooning degeneration in liver tissue and blocked the increase of adipocyte size in adipose tissue from treated *ob/ob* mice. These results indicated that the extract of key components in the traditional Chinese prescription MTF continue a potent anti-adipogenic and glucose-lowering agent.

## 1. Introduction

Diabetes mellitus (DM) is a chronic, progressive, incurable metabolic disorder characterized by hyperglycemia due to defective insulin action, insulin secretion or both [1]. The long-term elevated blood glucose level can easily cause a variety of diabetic complications such as ketoacidosis [2], neuropathy [3], nephropathy [4], cardiopathy [5] and retinopathy [6]. With the total number of diabetics projected to rise from 171 million in 2000 to 366 million in 2030 [7], DM has become a serious problem which is threatening world public health [8], therefore, various methods for the treatment of DM have been developed to maintain normal blood glucose or insulin levels.

Several drugs, including insulin, tolbutamide, and metformin, are commonly used in the clinic to treat diabetes, and the majority are chemical agents [9]. Several of these chemical drugs have unacceptable side effects in some patients. As a result numerous studies have focused on the anti-diabetic functions of Chinese herbs, which have demonstrated good results and shown a bright future in the therapy of diabetes. Numerous Chinese herbs have been used traditionally for the treatment of diabetes [10], including milkvetch root, salviae miltiorrhizae, and mulberry, which are key components of a Chinese herbal combination called Mai Tong Fang (MTF, Table 1).

**Table 1:** The key ingredients in the extract of MTF.

Common Name	Voucher Specimen Number	Drug Name	Latin Name	Family
Milkvetch root	No. 405	Radix Astragali	*Astragalus membranaceus (Fisch.) Bunge*	Leguminosae
Salviae miltiorrhizae	No. 1101	Radix Salviae Miltiorrhzae	*Salvia miltiorrhiza Bunge*	Labiatae
Mulberry	No. 476	Fructus Mori	*Morus alba L.*	Moraceae

Milkvetch root was reported to regulate the level of blood glucose and to be a potential treatment for diabetic nephropathy [11,12,13,14,15]. Similarly, mulberry has been proven to exhibit a marked blood glucose lowering effect in normal and alloxan-induced diabetic mice at low doses [16]. Salviae miltiorrhizae is also an important Chinese herb used to promote blood circulation [17]. The extract of milkvetch root, salviae miltiorrhizae, and mulberry in the prescription of MTF improved glucose metabolism, inhibited platelet aggregation and regulated vasomotor function in alloxan-induced diabetic rats [18]. Importantly, this mixture reduced urinary protein, improved lipid metabolism and blood viscosity in treating diabetic nephropathy in the clinic [19]. However, the anti-adipogenic and anti-hyperglycemic effects of the extract of milkvetch root, salviae miltiorrhizae and mulberry (MTF) have not been fully elucidated, so this study explored these pharmacological activities in 3T3-L1 adipocytes and animal model of type II diabetes (*ob/ob* mice), respectively.

## 2. Results

### 2.1. TG Accumulation in 3T3-L1 Adipocytes 

**Figure 1:**
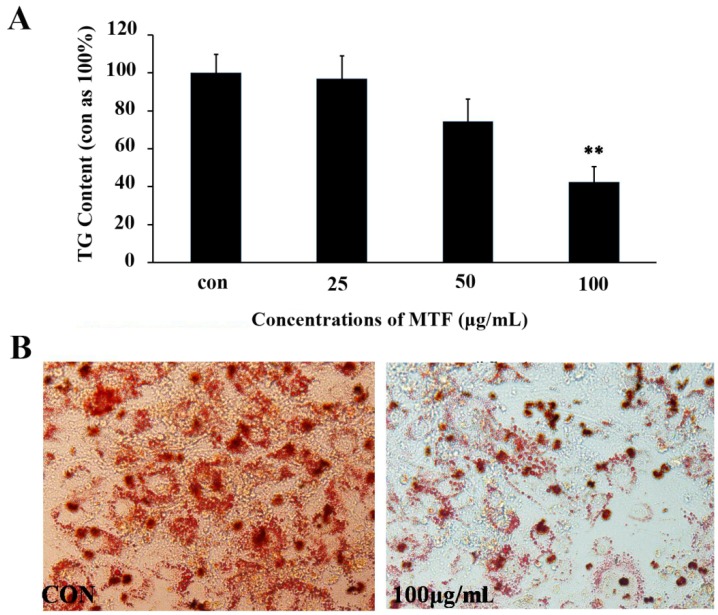
The MTF repression of intracellular lipid accumulation in 3T3-L1 cells. (**A**) Cells were treated with MTF at different concentrations during days 7–12 in the differentiation medium. On day 12, cells were lysed, and the intracellular TG level was analyzed; (**B**) Oil Red O staining of MTF-treated (100 μg/mL) 3T3-L1 cells. Data are presented as the mean ± SD from three independent experiments. ** *p* < 0.01 *vs.* control.

To confirm whether MTF is effective in inhibiting TG accumulation in 3T3-L1 cells, various concentrations of MTF were evaluated. As compared with control group, MTF effectively suppressed fat deposition in 3T3-L1 cells in a dose-dependent manner. When the concentration was 100 μg/mL, the rate of TG inhibition reached a maximum value of approximately 58% (from 3.07 ± 0.25 nmol/L to 1.29 ± 0.18 nmol/L, *p* < 0.01; Figure 1A). At the same time, the decrease of lipid droplet could be observed by Oil red O staining (Figure 1B).

**Figure 2:**
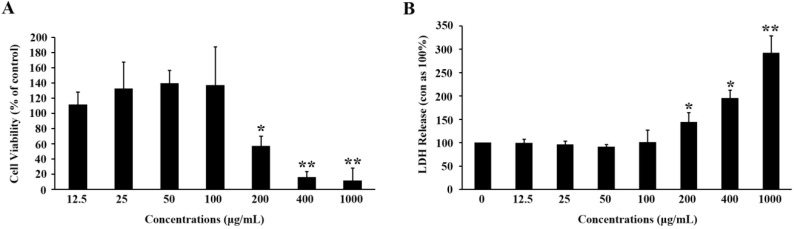
Cell toxicity of MTF on 3T3-L1 adipocytes. (**A**) Cells were incubated with various concentrations of MTF for 48 h. Cell toxicity was then measured in terms of cell viability; (**B**) The cell medium was collected 6 days after the incubation with MTF. LDH release was determined by its activity in the medium and corrected with the control. Data are mean ± SD from three independent experiments. * *p* < 0.05; ** *p* < 0.01 *vs.* control.

To exclude a possibility of the decrease in TG content resulting from cytotoxicity, we determined the MTT and LDH activity (Figure 2). After the experiments, we found that MTF displayed cell toxicity when the concentration was up to 200 μg/mL. According to the analysis, MTF exhibited a potent lipid-lowering effect that was not related to any cytotoxicity.

### 2.2. Body Weight and Food Intake

**Figure 3:**
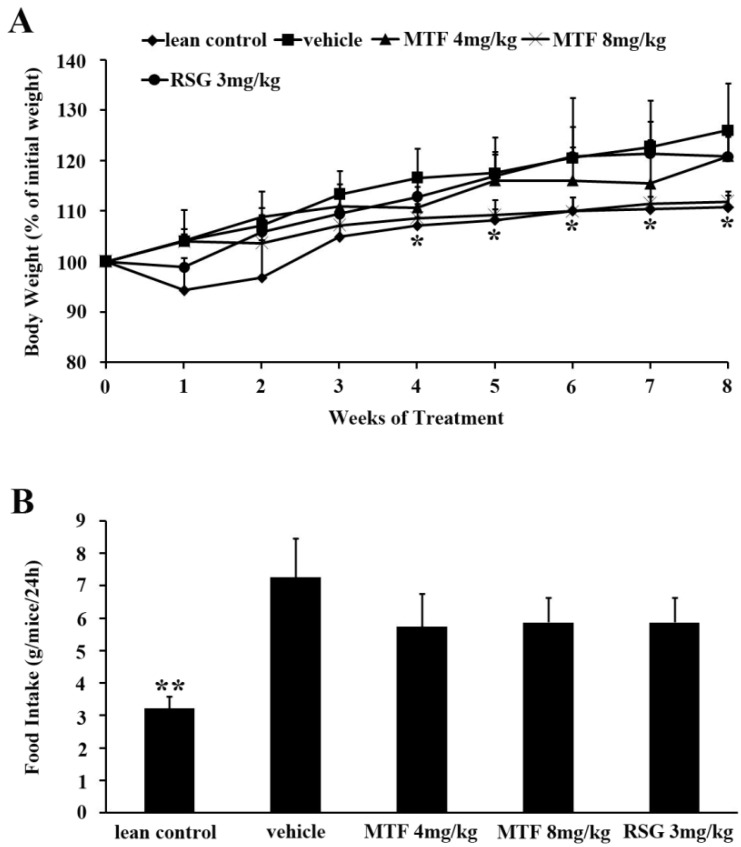
Body weight and food intake: (**A**) Changes of body weights in each group over the test period; (**B**) The food intake was recorded everyday during the study. Results are expressed as the mean ± SD (*n* = 8). * *p* < 0.05; ** *p* < 0.01 *vs.* vehicle.

Body weight and food intake of *ob/ob* mice are depicted in Figure 3. The untreated obese diabetic mice showed a significant higher body weight compared to that of lean controls. The extract of MTF limited the increase of body weight in a dose-dependent manner without affecting food intake. However, rosiglitazone (RSG) did not reduce body weight. 

### 2.3. Oral Glucose Tolerance Test (OGTT) and Insulin Tolerance Test (ITT) 

**Figure 4:**
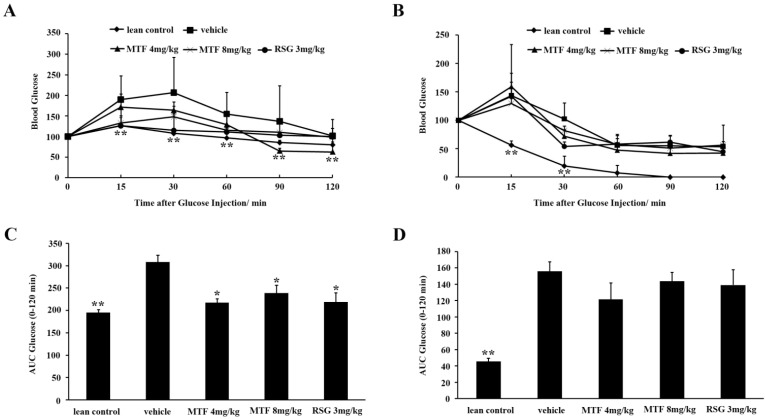
OGTT and ITT in *ob/ob* mice: (**A**) After 10 h of fasting, blood glucose concentrations were measured at the indicated times following 2 g/kg glucose was gavaged into mice; (**B**) After 10 h of fasting, 0.75 U/kg of insulin was injected intraperitioneally into mice. Blood glucose levels were measured at the indicated times shown in the graph; AUCs for (**C**) OGTT and (**D**) ITT are shown in the graph. Values are the mean ± SD (*n* = 8). * *p* < 0.05; ** *p* < 0.01 *vs.* vehicle.

As shown in Figure 4A, MTF extract significantly reduced blood glucose levels at all time points from 15 to 120 min by OGTT (*p* < 0.05). Both MTF and RSG lowered glucose levels when compared with the untreated *ob/ob* mice. The AUC in both MTF- and RSG-treated mice were decreased in contrast to vehicle (*p* < 0.05, Figure 4C). Hence, the extract of three Chinese herbs milkvetch root, salviae miltiorrhizae and mulberry is efficacious in ameliorating obesity-related diabetic symptoms. However, after the treatment, the changes of glucose and AUC in the ITT test were not obvious compared to vehicle group (*p* > 0.05), implying that MTF was weak in regulation of insulin resistance (Figure 4B,D). 

### 2.4. Serum Biomarkers

The serum level of glucose was significantly increased in vehicle as compared to lean control (*p* < 0.01). After 8 weeks of MTF and RSG treatments in *ob/ob* mice, the serum glucose levels were lower than that of the vehicle group (*p* < 0.01). Administration of MTF and RSG both exhibited evident hypoglycemic effects. In addition, the serum levels of TG (*p* < 0.01), TC (*p* < 0.01), and ALT (*p* < 0.01) were markedly higher in *ob/ob* mice than those of lean mice. In the measurement of serum TC, TG and ALT, both MTF and RSG down-regulated TC level (*p* < 0.05) after 8 weeks of treatment. However, treated with MTF at 8 mg/kg reduced the serum TG (*p* < 0.05) of *ob/ob* mice, while no evident change in the RSG-treated mice. As for the regulation of ALT level, RSG group reached a plateau. Compared to RSG, the serum of ALT level (*p* < 0.01) was lowered by the MTF treatment. Furthermore, MTF slightly changed the concentration of HDL-c and LDL-c in plasma but there was no significant difference compared to vehicle (Table 2). In the next work, we will further evaluate the lipid biomarker levels and the insulin resistance of other tissues to understand and clarify these results.

**Table 2:** Serum parameters of *ob/ob* mice.

Parameter	Vehicle	MTF 4 mg/kg	MTF 8 mg/kg	RSG 3 mg/kg
GLU (mM)	23.94 ± 11.77	15.67 ± 3.17 **	12.48 ± 1.88 **	11.30 ± 3.46 **
TG (mM)	1.57 ± 0.13	1.33 ± 0.34	1.14 ± 0.07 *	1.58 ± 0.20
TC (mM)	8.27 ± 0.96	5.57 ± 0.63 *	5.30 ± 1.24 *	4.46 ± 1.05 *
ALT (mM)	220.0 ± 116.8	304.7 ± 75.1 **	303.0 ± 36.3 **	987.4 ± 160.4 **
HDL-c (mM)	1.27 ± 0.32	1.42 ± 0.18	1.14 ± 0.27	2.16 ± 0.28
LDL-c (mM)	0.35 ± 0.09	0.26 ± 0.10	0.31 ± 0.08	0.57 ± 0.08

Results were expressed as mean ± SD (*n* = 8). * *p* < 0.05; ** *p* < 0.01 *vs.* vehicle.

### 2.5. Evaluation of Liver and Adipose Histopathology

**Figure 5:**
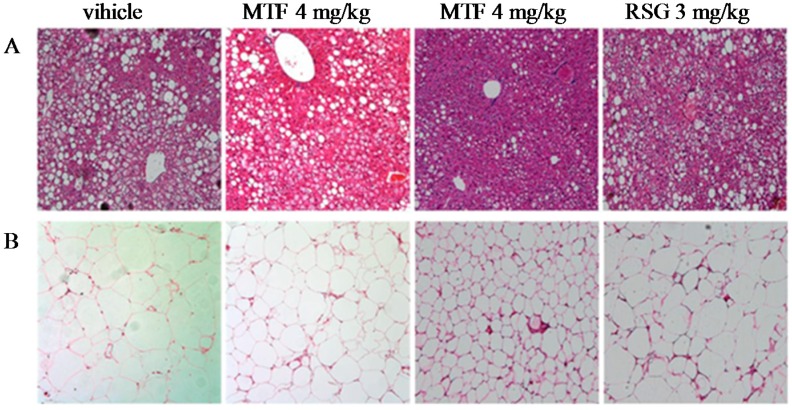
H&E staining of the (**A**) liver and (**B**) white adipose tissues from the vehicle-, MTF-, and RSG-treated *ob/ob* mice.

Photomicrographs of liver sections stained by H&E are shown in Figure 5A. Liver sections from mice in the lean control group displayed no obvious histological changes. However, remarkable hepatic lipid accumulation was found in non-treated *ob/ob* mice. Fat deposition produced severely damaged liver histology which presented as ballooning degeneration. After treatment with MTF, liver histological evaluations were normalized, which was confirmed by the significant improvement of steatosis and ballooning degeneration. Furthermore, in *ob/ob* mice, the volume of adipocytes in adipose tissues evidently increased. Through the MTF treatment, the size of adipocytes was significantly smaller than those of vehicle group, while RSG had a weaker effect on the adipose tissues (Figure 5B).

## 3. Discussion 

The hallmark of adipocyte differentiation is the formation and enlargement of intracellular lipid droplets [20]. In this study, the extract of three herbs milkvetch root, salviae miltiorrhizae and mulberry as key components of MTF effectively decreased the accumulation of lipids in 3T3-L1 cells. Meanwhile, the inhibition of TG could also observed by Oil Red O staining.

The increase of body weight corresponding to hyperglycemia was more pronounced in *ob/ob* mice. Also, levels of serum TG and TC were higher in *ob/ob* diabetic mice than those of the non-diabetic group. In the present study, 8-week treatment with MTF attenuated serum glucose concentration as well as ameliorated the increase of body weight in *ob/ob* mice. Excess body fat in obese individuals appeared to play a role in insulin resistance, which causes diabetic complications such as heart disease, hypertension, stroke, and unhealthy cholesterol levels. Therefore, combinative therapeutic methods for the treatment of diabetes needed to include diabetic dyslipidemia [21]. Although there was no significant difference compared to vehicle, MTF slightly modulated the serum levels of HDL-c and LDL-c and the results were in accordance with the level of TG and TC. In the light of this, the well-modulated serum parameters in *ob/ob* mice indicated that MTF might prevent the development and progression of type 2 diabetes and dyslipidemia.

Determination of glucose or insulin AUC values for the OGTT or ITT represent a simple method that has widespread used [22]. The glucose and insulin AUCs were an indirect index of *in vivo* peripheral insulin action and have been used to assess insulin sensitivity in animals [23] and humans [24]. AUC was therefore measured in the present study to ascertain the effect of MTF on glucose tolerance and insulin sensitivity. The results showed that MTF significantly decreased the AUC values for the OGTT, which highlighted that MTF regulated the glucose tolerance of *ob/ob* mice.

To evaluate whether long-term administration of MTF was toxic to liver tissues, serum ALT was assessed at the end of the experiment. ALT are enzymes that become elevated with liver disease or injury. The *ob/ob* mice, with or without MTF, had similar levels of ALT, indicating that chronic treatment with MTF did not have any appreciable toxic effects on the liver.

## 4. Experimental Section

### 4.1. Preparation of Herbal Extracts

Milkvetch root, salviae miltiorrhizae and mulberry as key components of MTF were produced by Sunnyhope Pharmaceutical Co., Ltd. (Chengdu, China) under internationally certified GMP guidelines. Individual herbs (in 1000 g gross weight, milkvetch root, salvia miltiorrhizae and mulberry are 200 g, 350 g and 450 g, respectively) were mixed and boiled twice with water (1 L × 2) at 100 °C and filtered. The filtered solution containing the extract was evaporated. This extract was taken up into ethanol, mixed up evenly and the ethanol was evaporated under reduced pressure until the smell of ethanol disappeared. The extract (designated as MTF in the study) was obtained after filtration.

### 4.2. HPLC-MS Analysis

**Figure 6:**
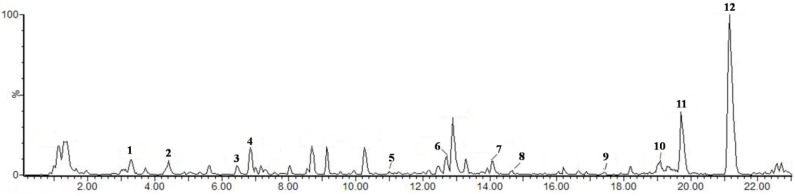
HPLC-MS chromatograms of MTF.

HPLC-MS was performed with an ACQUITY UPLC and QuattroPremier XE MS system. Chromatography of 10 μL injections was performed on an ACQUITY UPLC column (100 mm × 2.1 mm I.D. 1.7 μm) with a column temperature of 25 °C. A gradient elution of A (1% aqueous formic acid) and B (methanol) was used starting with and maintaining 90% A and 10% B for 2 min, then 10% A and 90% B for 25 min. The flow-rate of the mobile phase was 1.0 mL/min. Detection wavelength was 280 nm. The mass spectra were recorded using ESI in the negative mode with ion spray voltage set at 2.8 kV, source temperature at 100 °C, cone and desolvation gas flow 40 and 600 L/h and scanning from 100 to 1000 *amu*. Figure 6 shows the fingerprint of MTF and 12 peaks in MTF that were separated and identified by HPLC-MS (Table 3). 

**Table 3:** HPLC-MS preliminary analysis of MTF.

Peak	Predicted Compound	MW	[M−H]^−^	Milkvetch Root	Salviae Miltiorrhizae	Mulberry
1	Danshensu	198	197		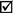	
2	Formononetin-7-O-β-gluco-pyranoside	516	515	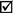		
3	Cafleoylquinic acid	354	353			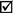
4	Unknown		375			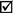
5	Salvianolic acid J	537	536		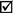	
6	Rosmarimic acid	360	359		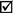	
7	Salvianolic acid A	494	493		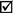	
8	Calycosin	284	283	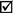		
9	Formononetin	268	267	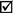		
10	Carnosol	330	329	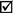		
11	Asp	515	514			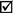
12	Val	499	498			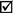

### 4.3. Cell Culture 

3T3-L1 mouse embryonic fibroblasts (ATCC, Manassas, VA, USA) were cultured in Dulbecco’s modified Eagles’ medium (DMEM) supplemented with 10% (v/v) fetal bovine serum (FBS) at 37 °C in 5% CO_2_. They were differentiated into adipocytes as described [25]. Briefly, 3T3-L1 pre-adipocytes were seeded into 96-well plates and allowed to reach full confluence. Two days after confluence, cells were incubated in DMEM containing 10% FBS, 5 μg/mL insulin (Sigma-Aldrich, Melbourne, Australia), 0.5 mM IBMX (Sigma) and 0.25 mM dexamethasone (Sigma) to induce differentiation (day 0, d0). Three days later (d3), the medium was changed to DMEM with 10% FBS and 5 μg/mL insulin for additional three days (d6). To assess the effects on lipid accumulation, cells were treated with different concentrations of MTF. They were added to the medium from d7 to d12. Then, the intracellular TG level was analyzed by a commercial triglyceride quantification kit (BioVision, Milpitas, CA, USA) after cells were lysed.

### 4.4. Oil Red O Staining 

Oil Red O staining was used in combination with light microscopy to assess preadipocyte differentiation as preciously described [26]. Briefly, the treated differentiated 3T3-L1 cells were washed twice with ice-cold PBS buffer before staining with 0.5% Oil Red O in isopropanol for 30 min. Excess stains were then removed by rinsing the cells under water and then dried before microscopic examination.

### 4.5. Cytotoxicity Assay 

Cells were seeded in 96-well plates and allowed to attach overnight at 37 °C. They were then incubated with various concentrations of MTF for 48 h. At the end of treatment, cells were cultured with MTT at a final concentration of 0.5 mg/mL for 4 h. The purple MTT formazan was dissolved by DMSO, and the absorbance was quantified using a spectrophotometer at 570 nm. The cell viability (%) was obtained by comparing the absorbance of the samples and control. The cellular release of lactate dehydrogenase (LDH) was used as a measure of cellular damage. After cells were treated with MTF for 6 days in differentiation medium, the medium were collected. The enzymatic activity was determined by CytoTox 96 Non-Radioactive Cytotoxicity Assay kit (Promega, Melbourne, Australia) according to the manufacturer’s instructions.

### 4.6. Animals 

Male obese mice (C57BL/6J-ob/ob) and their lean controls (C57BL/6J-+/+) were purchased from Western China Experimental Animal Center at 4–5 weeks of age. They were housed under standard animal care conditions with a normal chow diet and free access to water. After acclimatization for 1 week, the obese mice were divided into four groups (*n* = 8) randomly. Then, they were orally administered once a day with the vehicle (0.9% normal saline), MTF (4 mg/kg and 8 mg/kg), and rosiglitazone (RSG, 3 mg/kg), as the positive group for 8 weeks. Body weight and food intake were recorded during the study period. After the experimental period, animals were sacrificed. Blood, liver and adipose tissues were collected for further studies. All animal experiments were performed under protocols approved by National Institutes of Health’s Guide for the Care and Use of Laboratory Animals [27].

### 4.7. Oral Glucose Tolerance Test (OGTT) and Insulin Tolerance Test (ITT) 

OGTT was performed to determine the effect of MTF on obese mice at week 8. Mice were fasted overnight (10 h, only plain water was supplied for all animals) and orally administered with glucose (2 g/kg). Tail vein blood samples were withdrawn without anesthesia before (0 min) and 15, 30, 60, 90 and 120 min after the administration of glucose. Blood glucose was monitored using a glucometer. As for ITT, the animals were fasted overnight (10 h), and then tail blood was collected before (0 min) and at 15, 30, 60, 90 and 120 min after intraperitoneal injection of insulin (0.75 U/kg). The area under the curve for serum glucose (AUC_glu_) was calculated for the OGTT or ITT using software DAS 2.0 (Mathematical Pharmacology Professional Committee of China, Shanghai, China).

### 4.8. Measurements of Serum Parameters in ob/ob Mice 

Serum levels of glucose (GLU), triglyceride (TG), total cholesterol (TC), alanine transaminase (ALT), low-density lipoprotein cholesterol (LDL-c), and high-density lipoprotein cholesterol (HDL-c) were measured with a Hitachi 7020 automatic analyzer (Hitachi, Tokyo, Japan).

### 4.9. Histopathological Analysis 

Tissue samples were fixed by inflation with 4% buffered paraformaldehyde solution. The tissues were then embedded in paraffin, after dehydration, cut into sections (5 μm), and stained with a hematoxylin-eosin (H&E) solution. All tissue samples were examined and imaged in a blinded fashion. Images were captured using an Olympus DP72 microscope and manager at a magnification of 100×.

### 4.10. Statistical Analysis

All results were analyzed using SPSS 16.0 software (SPSS Inc., Chicago, IL, USA). Statistical comparisons were assessed with a one-way ANOVA or Student’s t-test for each paired experiment. All values were expressed as means ± standard deviation (SD). *p* value less than 0.05 was regarded as a significant difference.

## 5. Conclusions

In this study, we analyzed 11 compounds by the HPLC-MS method. We speculate that carnosol (a phenolic compound), formononetin (an isoflavone) and salvianolic acids (phenolic compounds) could exhibit potential anti-oxidant and anti-inflammatory activity to improve obesity and diabetic metabolic development, so we will further investigate the biological activity of these chemical compounds for the treatment of metabolic diseases.

In summary, MTF exhibited potent and potential effects in reducing TG in 3T3-L1 adipocytes. Furthermore, oral treatment with MTF significantly reduced the gain of body weight and improved glucose tolerance, and serum metabolic biomarkers in *ob/ob* mice. The findings of this study are of merit in revealing, for the first time, that MTF represents an anti-diabetic herb drug combination with plasma glucose lowering and glucose tolerance improving effects in *ob/ob* mice. These compelling pharmacological profiles have confirmed that MTF is a potential anti-diabetic agent for the treatment of metabolic diseases.
